# A Case Report on an Open Fracture Dislocation Injury of the Proximal Phalanx of the Thumb Resulting from Playing Cricket

**DOI:** 10.5070/M5.52278

**Published:** 2026-04-30

**Authors:** Michael R Chiang, Morgan Kemerling, Rahul Pentaparthi, Avi Ruderman

**Affiliations:** * University of Texas Southwestern Medical Center, Department of Emergency Medicine, Dallas, TX; ^ University of Texas Southwestern Medical Center, School of Medicine, Dallas, TX

## Abstract

Open fractures represent high acuity injuries requiring rapid evaluation and management for optimal outcomes. In this report, we discuss a patient presenting with an open fracture of the proximal phalanx of the right thumb with interphalangeal (IP) joint dislocation. The patient reported that the injury occurred in the setting of trauma from catching a cricket ball, experiencing immediate severe pain and bleeding. Key interventions in the emergency department included rapid x-ray evaluation of the injury, administration of cefazolin and tetanus vaccine, pain control, covering the exposed bone in moist gauze, and involvement of the hand surgery team for bedside irrigation and reduction. He underwent open reduction and internal fixation of the fracture the next day. He was followed in the hand clinic and was doing well at the 12-week appointment. In summary, we report on an open fracture and dislocation of the proximal phalanx of the thumb secondary to a traumatic cricket injury. Early mobilization of the surgical team and adjunctive prophylaxis with cefazolin and updated tetanus were key measures in the emergency department for achieving optimal outcomes.

**Topics:**

Finger injury, hand injury, open fracture, dislocation, cricket, sports injuries.

**Figure:**
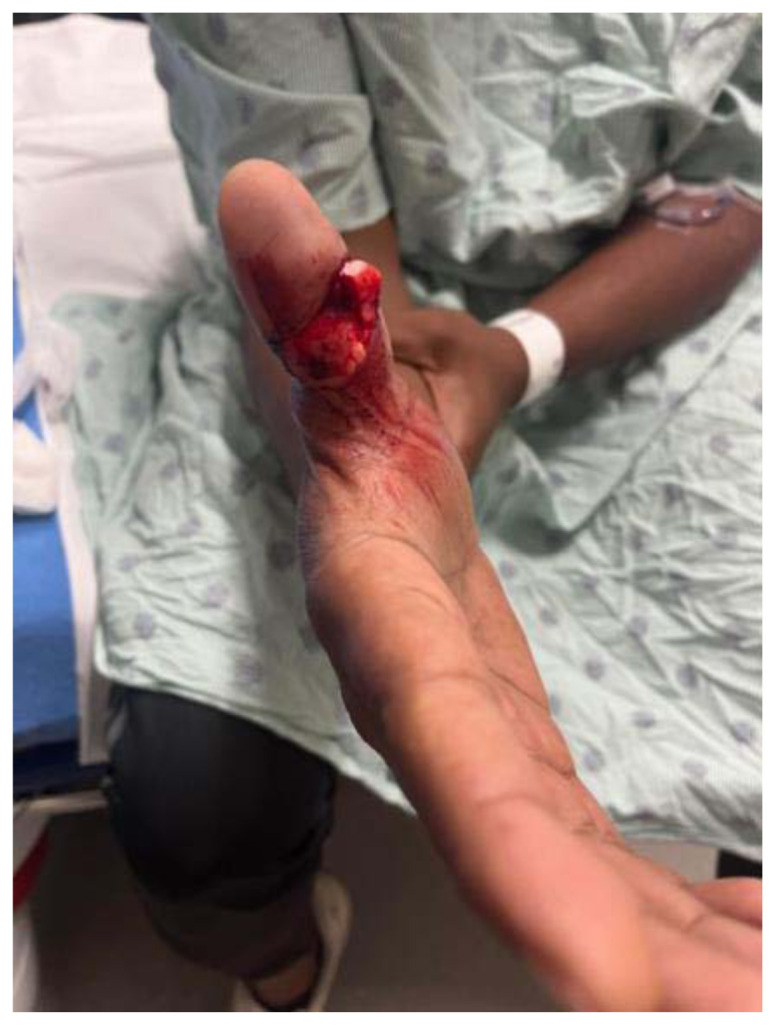


**Figure:**
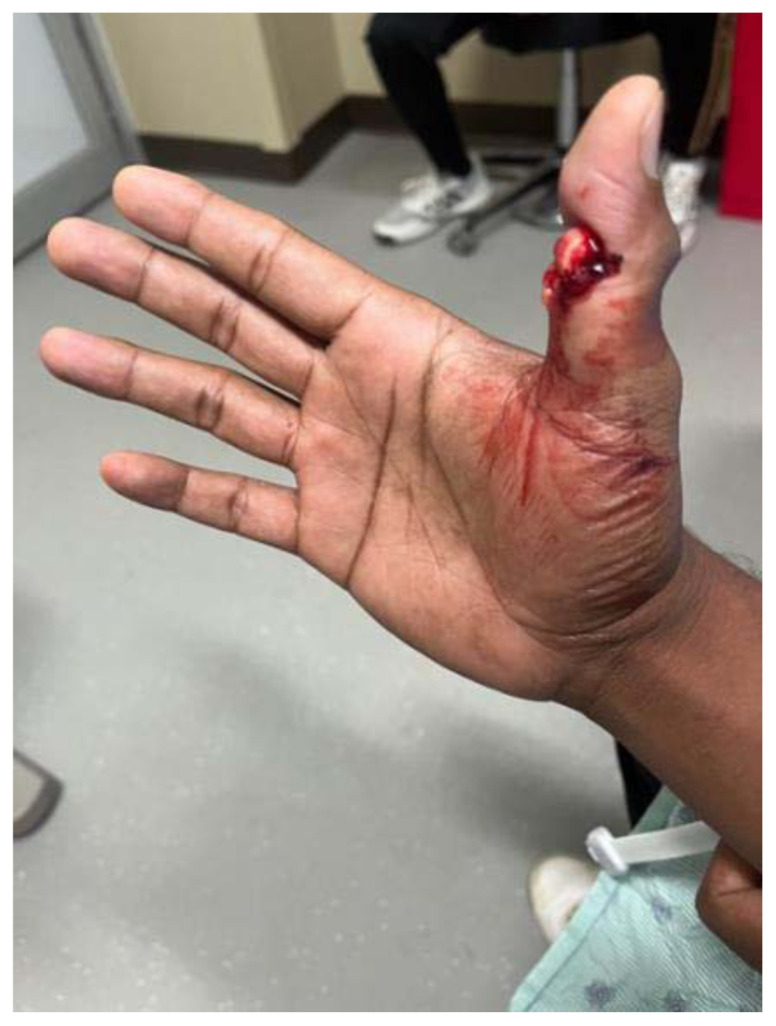


**Figure:**
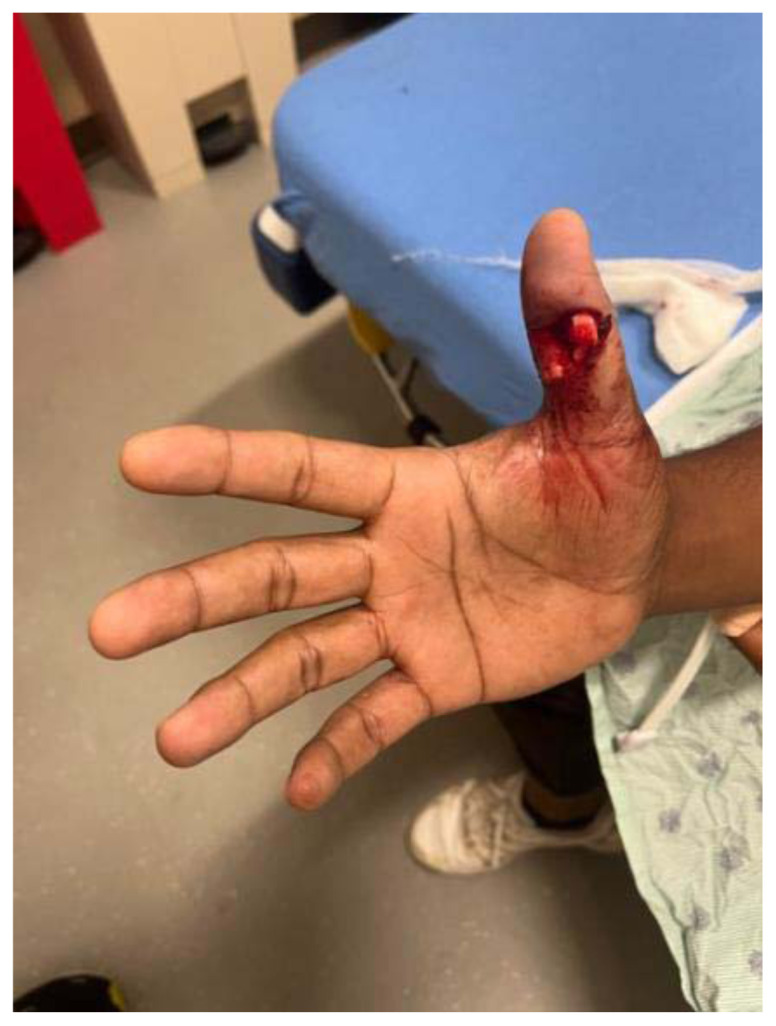


**Figure:**
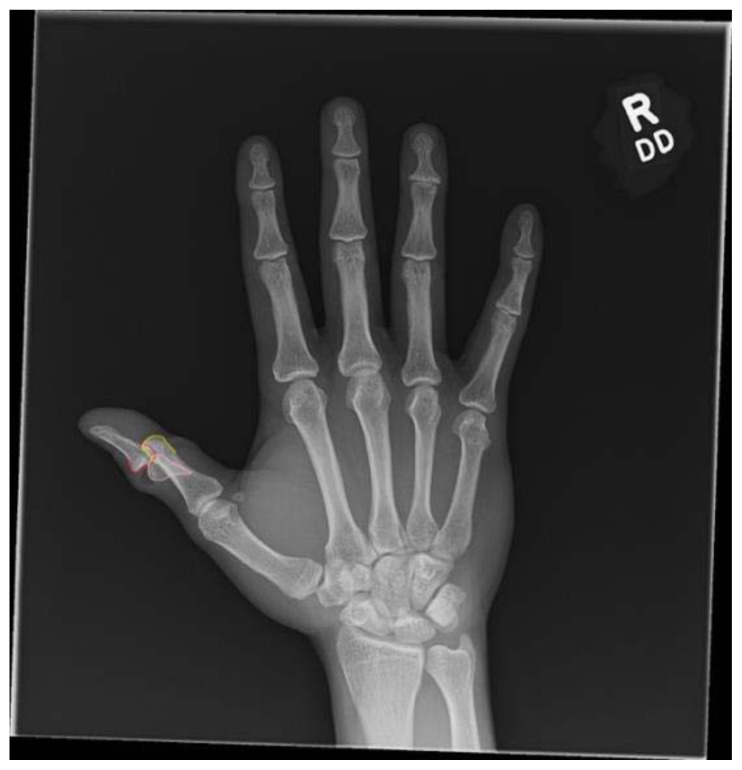


**Figure:**
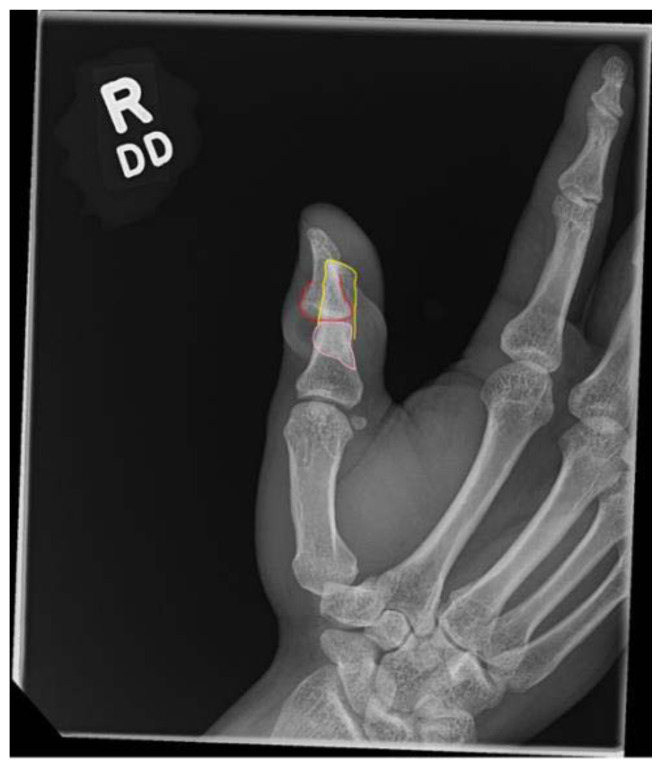


## Brief introduction

Open fractures of the thumb represent high acuity injuries. In the US, work-related trauma remains the primary cause of open hand fractures, with the small finger most commonly affected.^1^ Cricket is a sport that is growing in popularity in the United States. Played with a cork ball that is smaller, harder, and heavier than a baseball, cricket is played without hand padding, which contributes to increased hand injury patterns. In one study of 64 cricket-related hand injuries, 57 occurred while fielding, with 15 fracture-dislocations commonly affecting the phalanges and IP joints.^2^ Cricket players face significant risk of hand injuries, with studies showing a 14.5% to 18% annual incidence of injuries requiring absence from sport.^3^,^4^ Hand and wrist injuries are particularly prevalent, with fielding the ball being a common mechanism of injury.^5^–^8^ Surgical management options for hand fractures include wiring, pins, or screw and plate fixation techniques.^9^,^10^ Notably, antibiotic use and contamination level influence infection risk in open hand fractures, and these injuries generally carry a lower infection risk compared to other open fractures.^10^,^11^

## Presenting concerns and clinical findings

A 39-year-old, right-handed male with a past medical history of diabetes and hypertension presented to the Emergency Department (ED) approximately one hour after sustaining a right thumb injury while playing cricket. He reports that he was attempting to catch the ball, and the ball caused his thumb to bend backward and break. He reported immediate, severe pain and bleeding; however, the bleeding stopped within a few minutes of the injury. He denied numbness, tingling, or weakness of the right hand, as well as prior injuries to the right hand. Written consent was obtained for publication.

## Significant findings

There was an open injury to the volar aspect of the right thumb at the interphalangeal joint, with exposed bone. There was no active bleeding from the wound. He had intact sensation to the entire thumb and hand. His radial pulse was normal with normal capillary refill in all digits of his right hand. He had intact wrist flexion, extension, abduction, and adduction; however, he was unable to flex or extend his thumb secondary to the injury. He had no other injuries to the rest of his right upper extremity. An x-ray was obtained which showed a right thumb proximal phalanx intra-articular fracture (proximal fracture fragment outlined in yellow, distal fracture fragment outlined in pink) at the interphalangeal joint with dorsal dislocation of the distal phalanx (outlined in red). There were no radiopaque foreign bodies.

## Patient course

In the ED, the patient was given two grams of intravenous cefazolin, tetanus prophylaxis, and intravenous hydromorphone for pain control. After initial evaluation by the emergency medicine team, the hand surgery team was consulted for management and possible surgical intervention. The surgical team performed a bedside irrigation with normal saline after performing a digital block with lidocaine, and then attempted to reduce the fracture-dislocation at the bedside. The reduction was incomplete due to an incarcerated bone fragment. A thumb spica splint was placed and the patient was admitted to the surgical team and scheduled for the operating room (OR). The patient was discharged home the day of surgery with a splint in place and scheduled hand surgery follow up.

Approximately 12 weeks following surgery, x-rays demonstrated a completely healed fracture in anatomic positioning and physical exam showed an exceptionally well-healed incision with minimal scarring. After this appointment, the patient had no further activity restrictions and was allowed to play cricket again.

## Discussion

Although cricket is the second most popular sports game in the world with approximately 2.5 billion worldwide fans, healthcare professionals outside of the historical British commonwealth where it originated may be unfamiliar with the sport and its mechanisms of injury.^8^ Regarding impact related injuries from the cricket ball, the easiest comparison for US healthcare professionals would be akin to that of baseball, which also uses a similarly sized ball. Nonetheless, key differences include that the cricket ball is generally a harder and heavier object, and cricket players who are in the field receiving the ball are not allowed to use gloves or other hand-related safety equipment when catching or receiving the ball.^12^ Unlike cricket, baseball players wear mitts which offer additional padding in protecting players’ hands. As a result, hand related injuries are common in cricket.^2^–^8^,^12^

This patient had a comminuted intraarticular fracture of the proximal phalanx of the thumb, dorsal dislocation of the distal phalanx and contamination with grass and dirt. Cricket players are already at increased risk for hand injury, and one study found increased odds of hand pain and osteoarthritis in cricket players who had a history of hand injury.^3^ Intraarticular fractures are notoriously difficult to treat with a high incidence of long-term complications such as stiffness, pain, and increased risk of severe degenerative changes.^9^ Often, fixation devices such as screws or Kirschner wires are needed for stability, realignment, and subsequent improvement in function and range of motion.^9^,^10^ Expedited surgery evaluation and operative intervention is often needed for preservation of function.^9^,^10^ Because this patient’s injury was to his dominant hand, loss of function in this digit would have a debilitating effect on quality of life due to the thumb’s integral role in gripping, pinching, grasping, and gross hand movements.^9^ Fortunately, this patient received appropriate and expedited care with timely administration of prophylactic antibiotics and urgent evaluation by the hand surgery service.

One weakness of this case report was in the reporting of this patient’s mechanism of injury. While it was understood that the injury occurred while the patient was playing cricket, it is unclear at what point in the game the injury occurred. For example, the injury pattern caused by being struck by a missed lob would likely produce a different injury pattern from being struck at close range by a line drive. Having a better understanding of the mechanism of injury would enable a provider to better contextualize a patient’s risk and degree of injury, especially within the prehospital or triage setting.^8^

In summary, cricket related hand injuries can be high impact injuries that require expedited evaluation by a hand specialist. The decreased familiarity of cricket in relation to other sports in the United States such as American football and basketball may hamper American healthcare workers from accurately assessing the possible severity of injury on initial presentation. Given the high risk of hand injuries that cricket players sustain as well as the increased risk of subsequent hand pain, loss of function, and future osteoarthritis, recognition of the severity of such injuries is imperative for the long-term prognosis of patients with cricket-related injuries.^4^,^7^,^8^

## Supplementary Information


















