# Supplementary figures and images for: A Case Report on an Open Fracture Dislocation Injury of the Proximal Phalanx of the Thumb Resulting from Playing Cricket
Source: J Educ Teach Emerg Med. 2026 Apr 30;11(2):V16–9. doi: 10.5070/M5.52278 (PMC13152385; doi:10.5070/M5.52278)

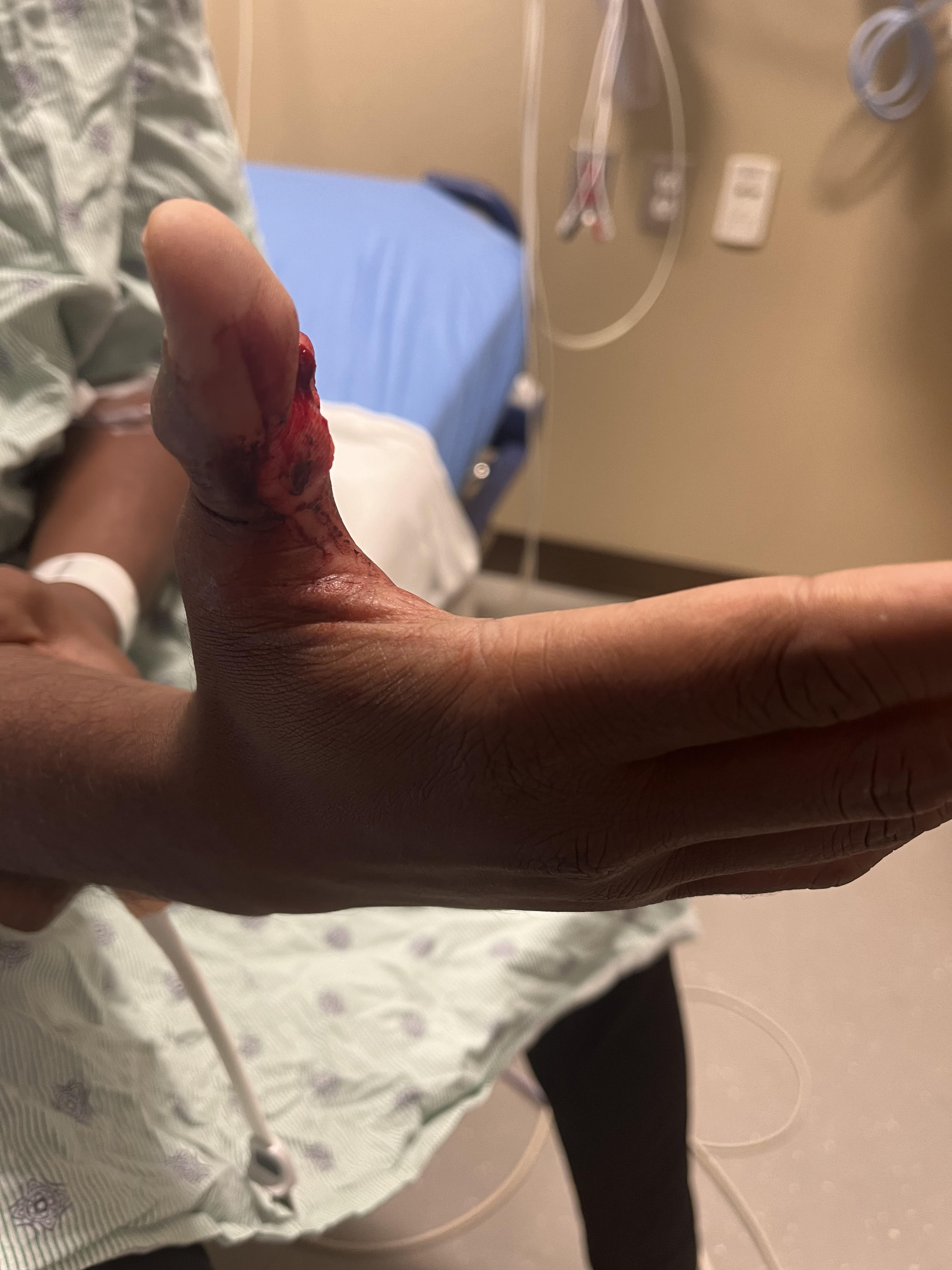

Supplement: Supplementary file 1 [file 11-2-V16-Supp1.jpg]

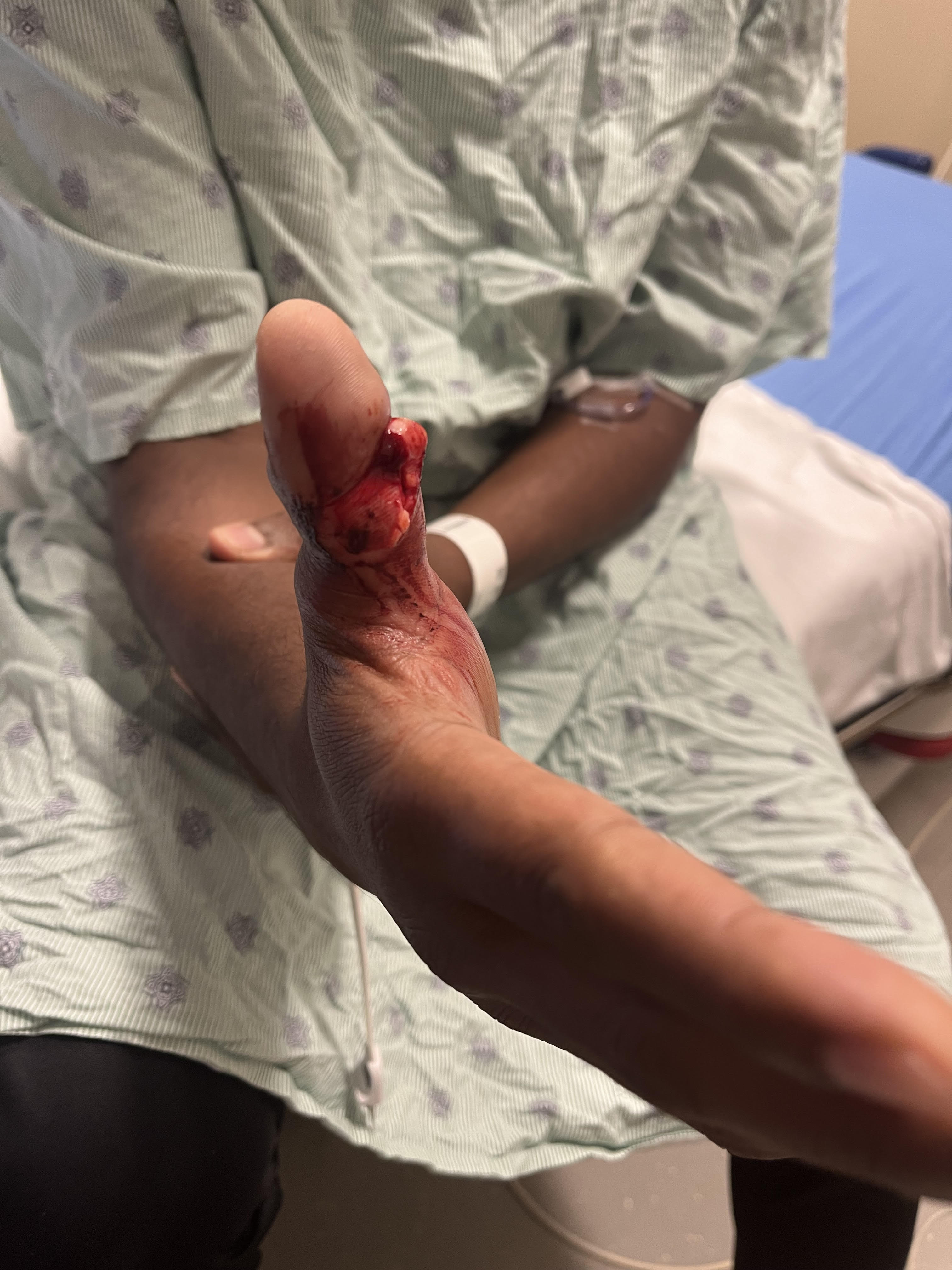

Supplement: Supplementary file 2 [file 11-2-V16-Supp2.jpg]

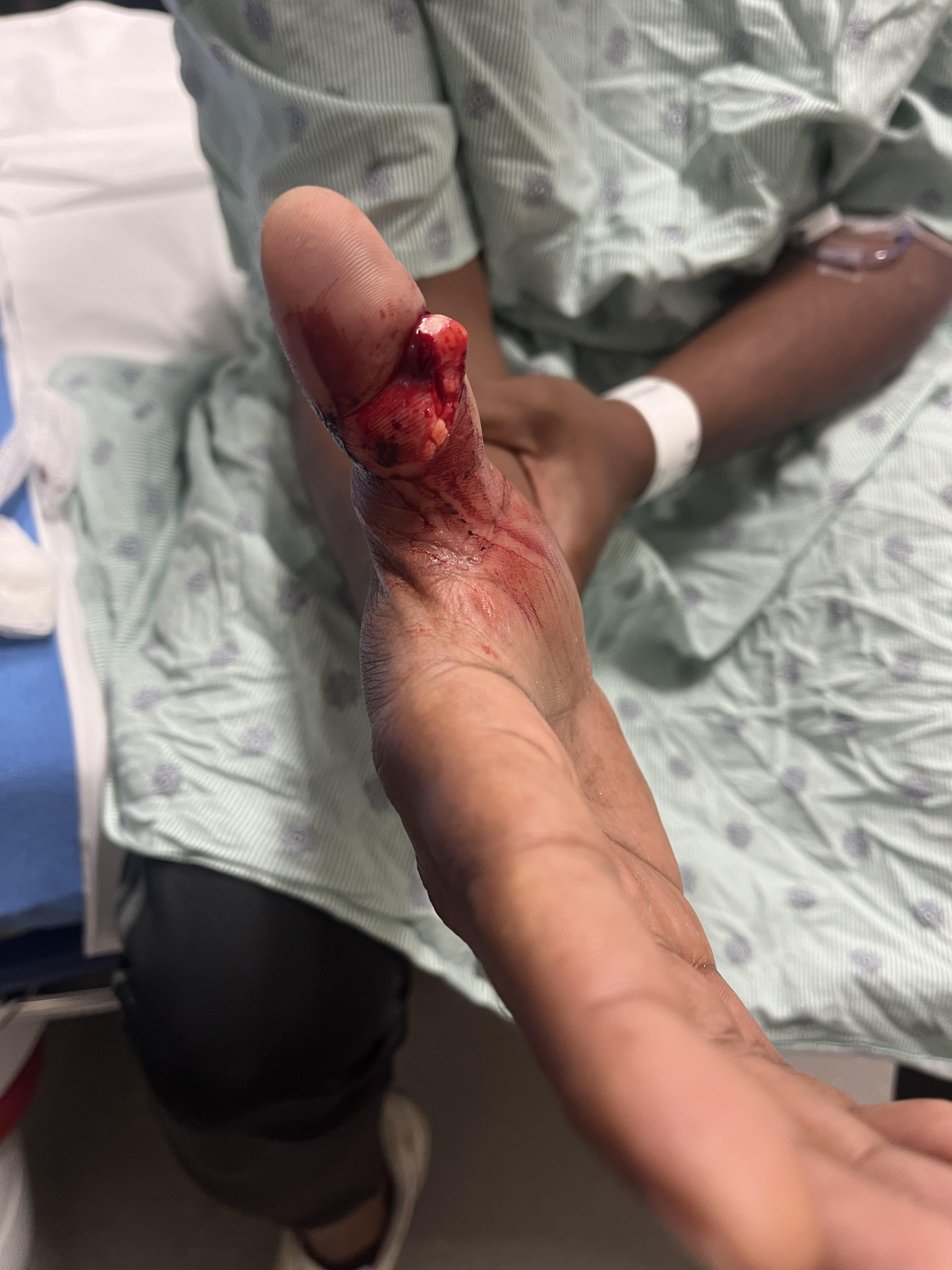

Supplement: Supplementary file 3 [file 11-2-V16-Supp3.jpg]

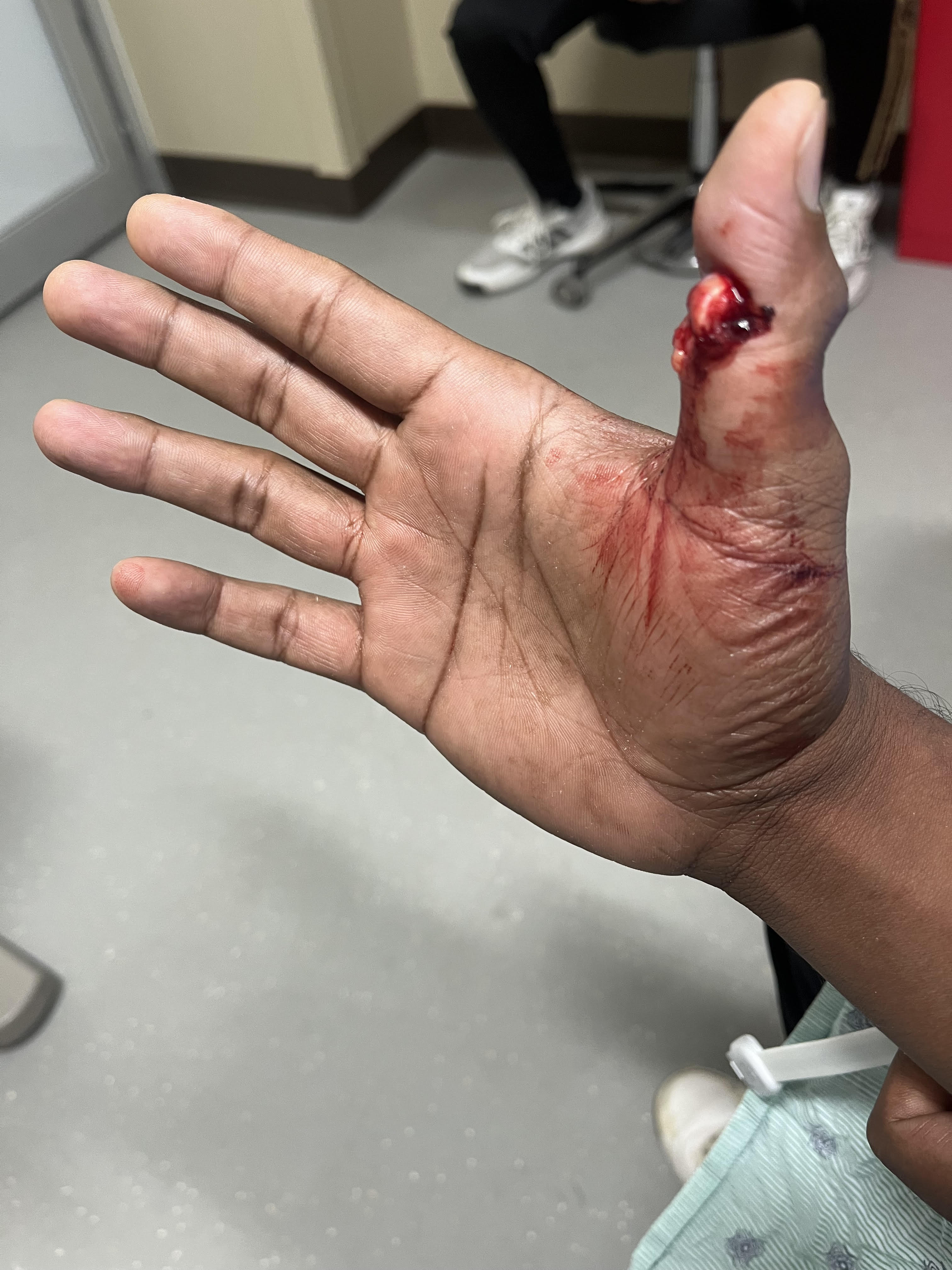

Supplement: Supplementary file 4 [file 11-2-V16-Supp4.jpg]

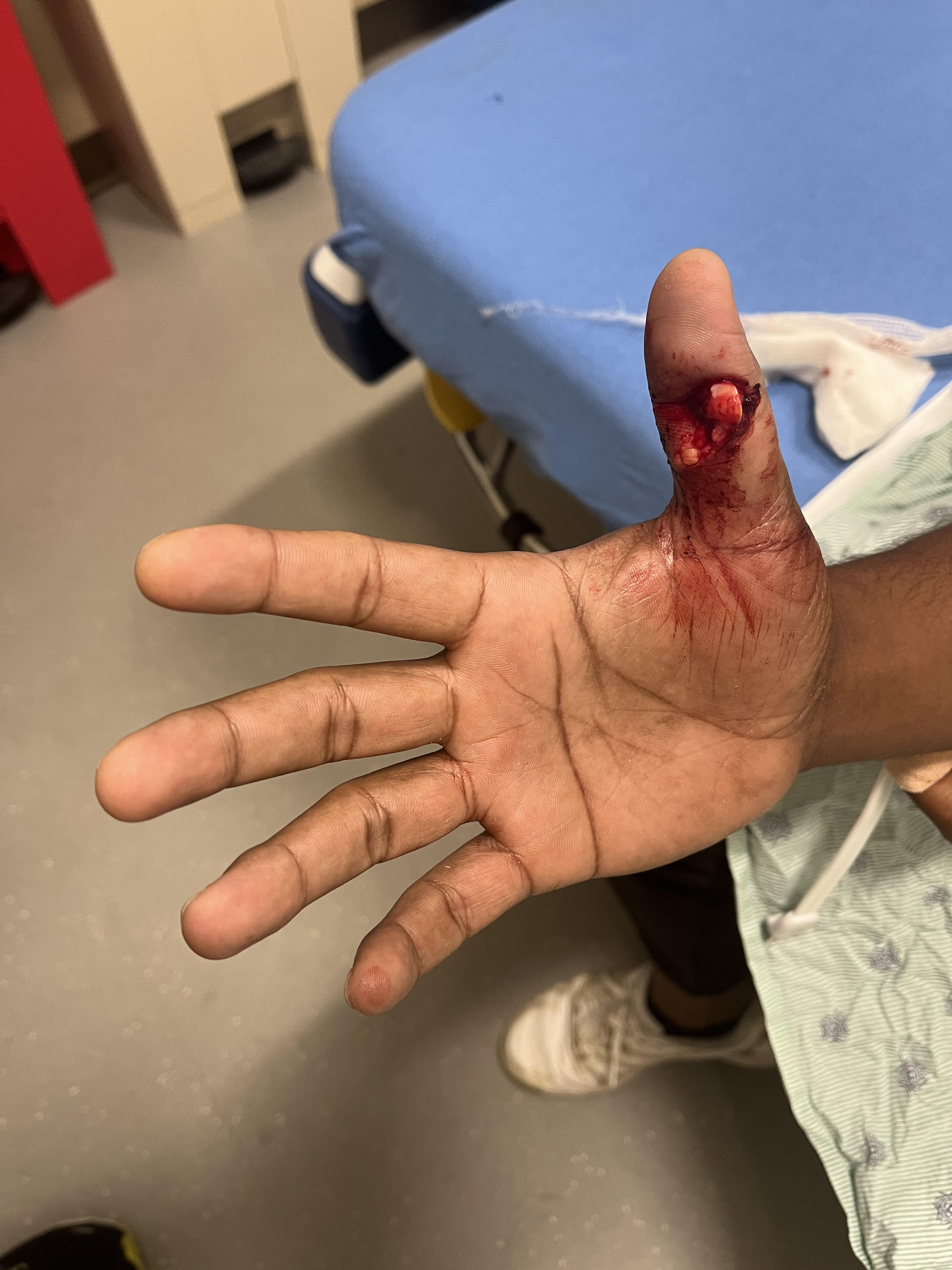

Supplement: Supplementary file 5 [file 11-2-V16-Supp5.jpg]

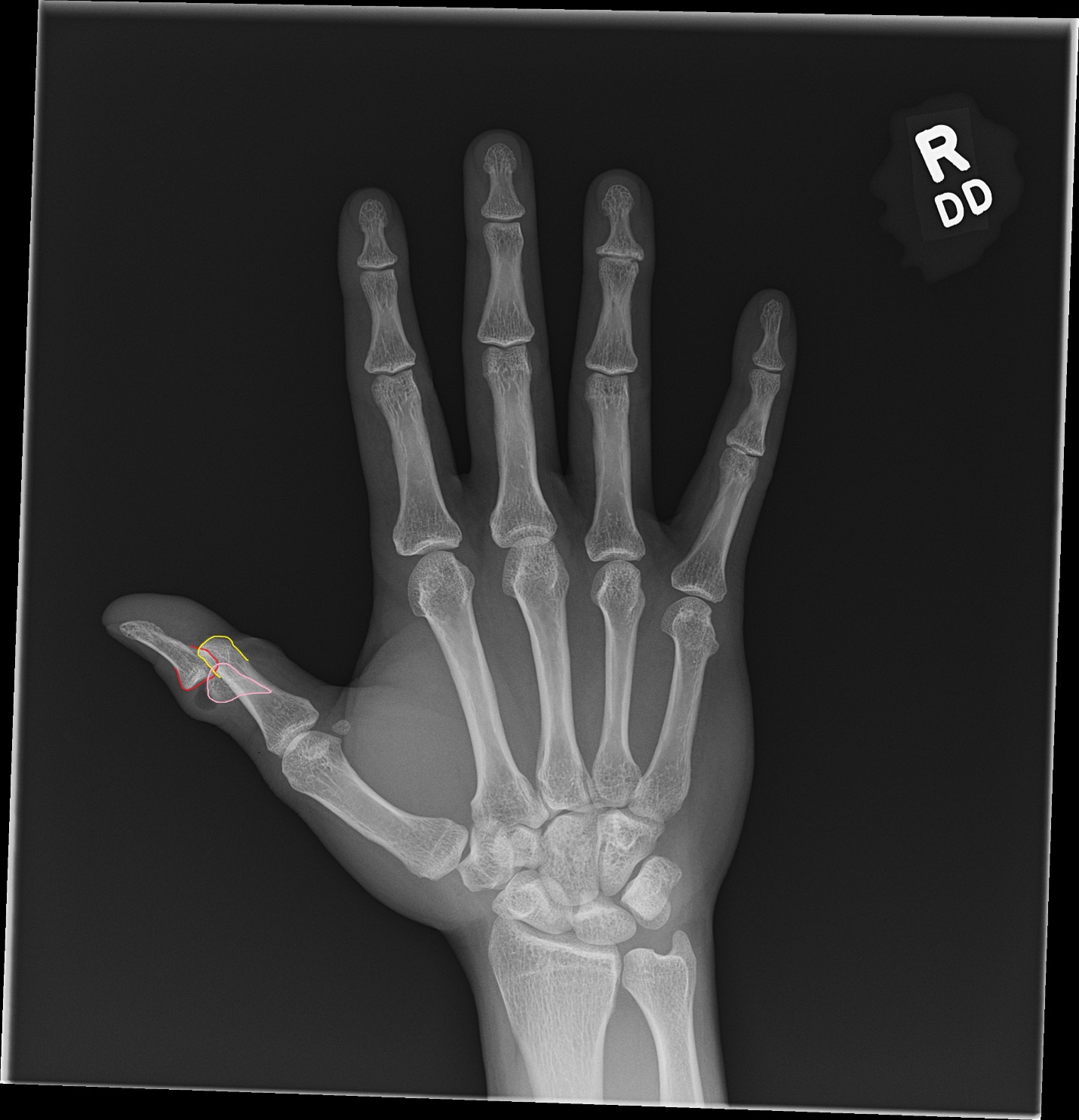

Supplement: Supplementary file 6 [file 11-2-V16-Supp6.jpg]

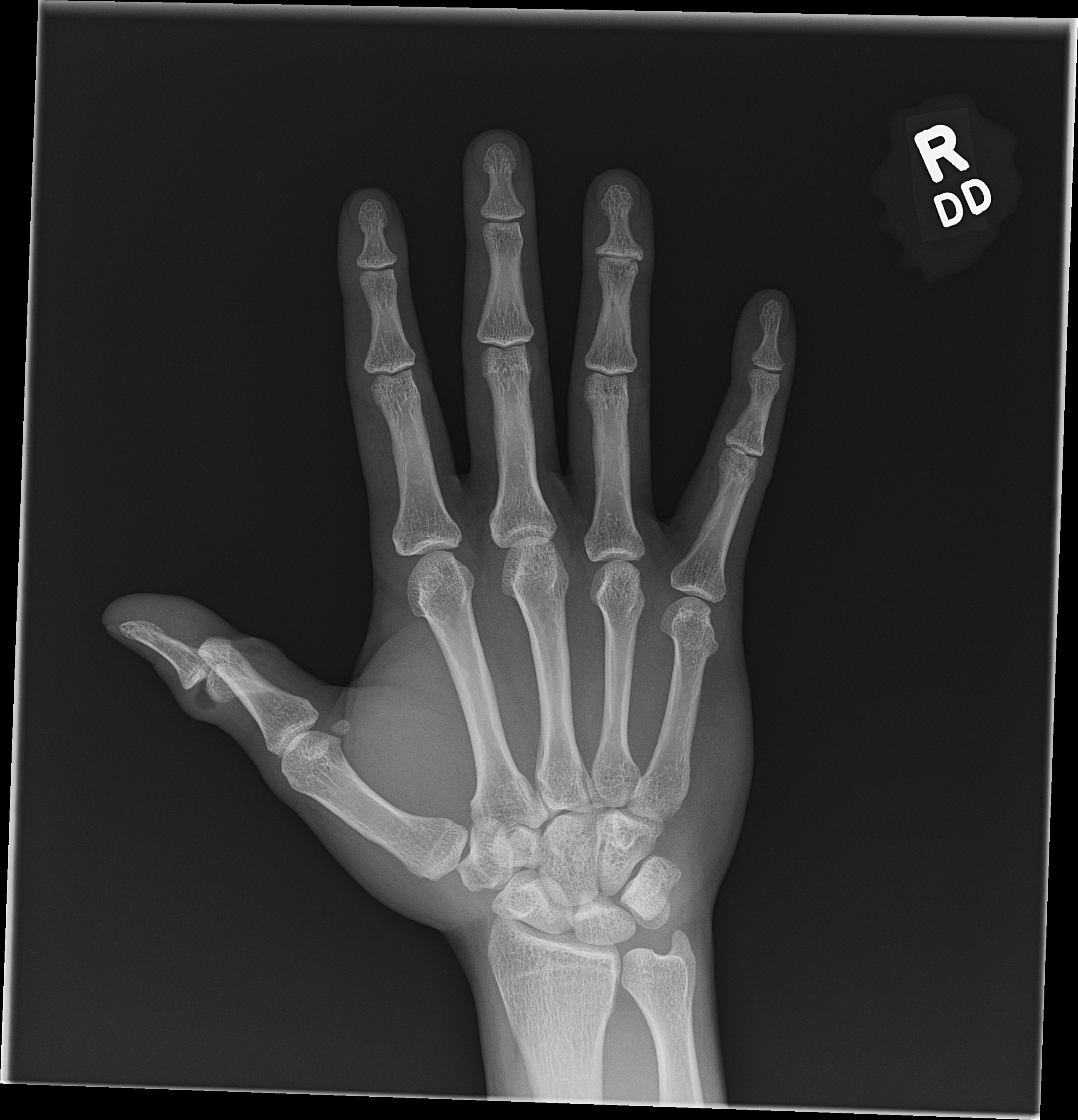

Supplement: Supplementary file 7 [file 11-2-V16-Supp7.jpeg]

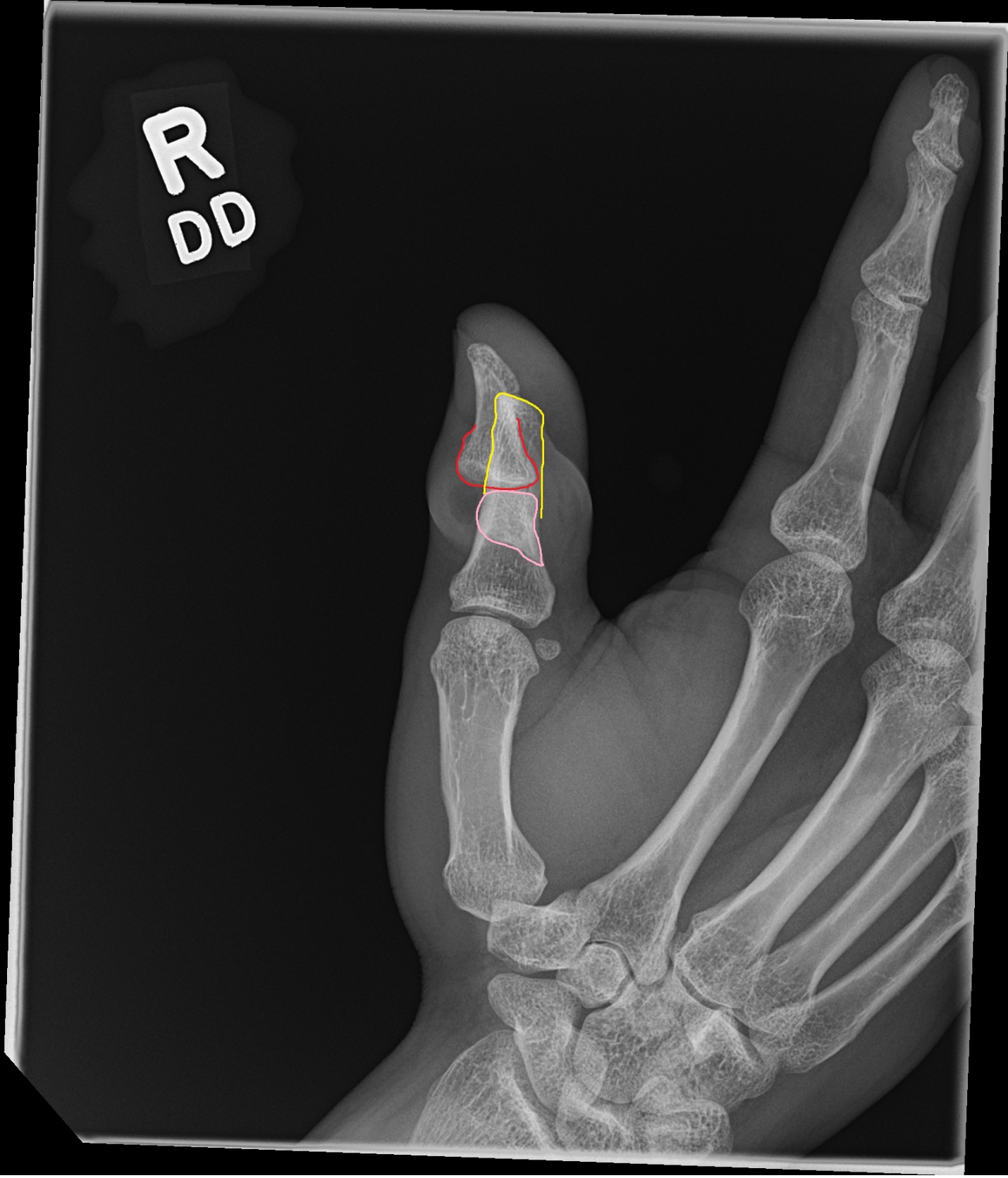

Supplement: Supplementary file 8 [file 11-2-V16-Supp8.jpg]

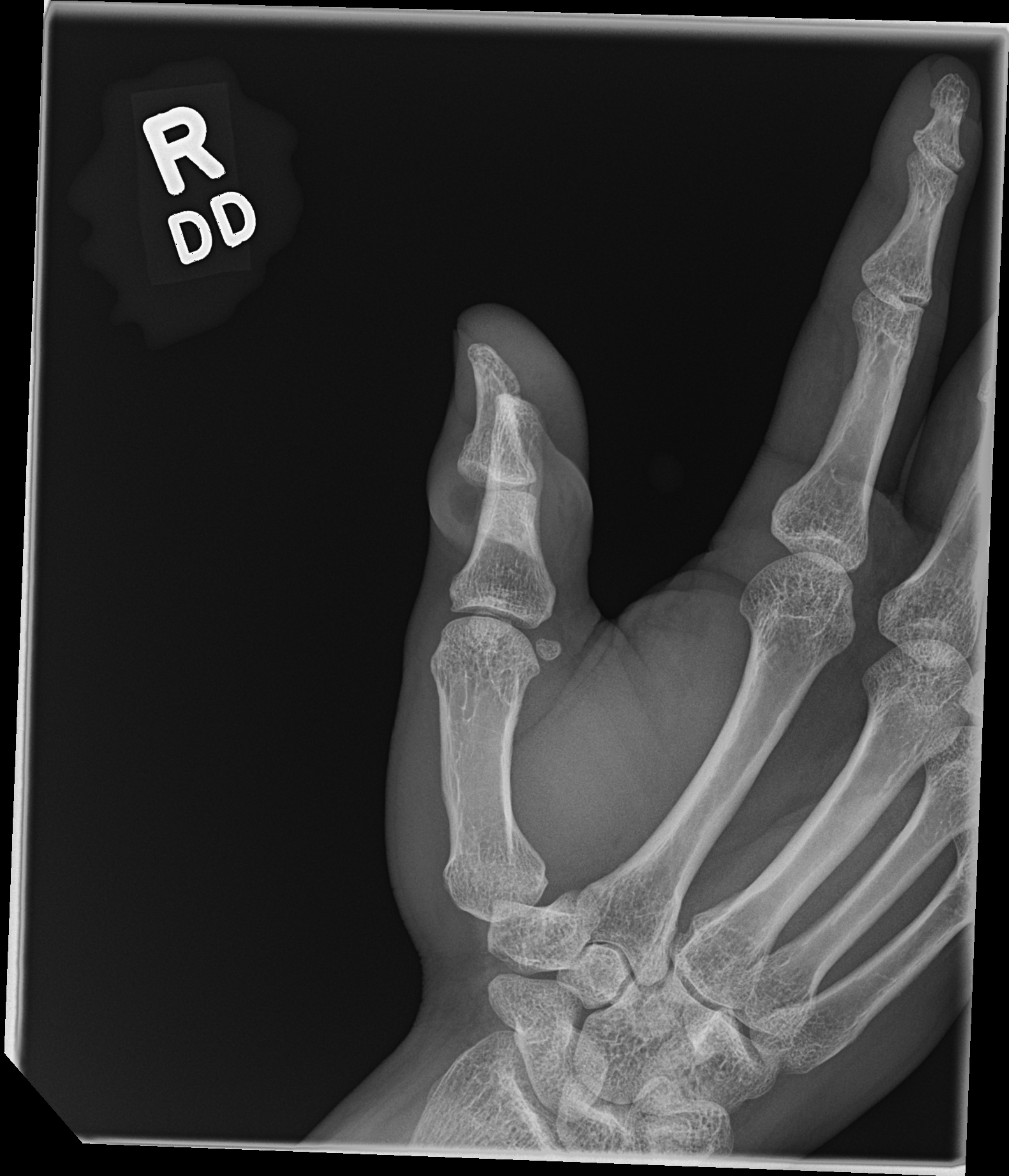

Supplement: Supplementary file 9 [file 11-2-V16-Supp9.jpeg]
